# The roles of cognitive and motivational imagery on flow: a multiple indicators multiple causes approach

**DOI:** 10.3389/fpsyg.2025.1729367

**Published:** 2026-01-05

**Authors:** Seungmin Lee, Jun-Phil Uhm

**Affiliations:** 1 Institute of Coaching, Waseda University, Tokyo, Japan; 2 Department of Kinesiology, Inha University, Incheon, Republic of Korea

**Keywords:** flow, imagery ability, mental rehearsal, multiple indicators multiple causes model, optimal experience

## Abstract

**Introduction:**

While mental training in coaching often targets Flow, it may overlook its multidimensional nature and distinct imagery pathways, limiting effective interventions for athletic performance. This study examined the effects of five imagery abilities—Skill, Strategy, Goal, Affect, and Mastery—on nine Flow dimensions in sport using a Multiple Indicators Multiple Causes (MIMIC) model.

**Method:**

Self-reported measures were obtained from 375 collegiate athletes, with confirmatory factor analyses supporting validity.

**Results:**

Initial MIMIC results indicated that motivational imagery abilities (Goal, Affect, Mastery) significantly predicted Flow (*β* = 0.08–0.28, *p*s < 0.05), whereas cognitive imagery (Skill, Strategy) showed no direct effects (*β* ≈ 0.00, *p*s > 0.05). A mediation model revealed that cognitive imagery was indirectly associated with Flow via motivational imagery across most dimensions, with effects ranging from *β* = 0.12 to 0.58 (*p*s < 0.016). Loss of self-consciousness exhibited weak, non-significant correlations, highlighting the difficulty of reproducing this component through mental rehearsal.

**Discussion:**

Findings suggest motivational imagery acts as a bridge between cognitive imagery and Flow, integrating motivational elements into cognitive-focused interventions to enhance Flow in competitive sport.

## 1 Introduction

Flow has garnered significant attention in sport psychology for its association with enhanced athletic performance, as athletes in flow states often report heightened concentration, effortless control, and peak performance levels (Harris et al., 2021; Jackson and Marsh, 1996). Athletes report unique bodily sensations during flow states, including altered perceptions of balance and arousal (Jackman et al., 2017). Since its conceptualization by Csikszentmihalyi (1990), Flow has been regarded as a central construct for understanding optimal psychological functioning in performance contexts. Beyond its immediate experiential qualities, Flow is closely linked to superior performance outcomes and heightened intrinsic motivation (Jackson and Marsh, 1996), making it a compelling focus for research on the cognitive and motivational mechanisms underlying peak states (Kawabata and Mallett, 2011).

One psychological skill frequently proposed to facilitate entry into Flow is imagery—the mental simulation of performance-relevant experiences (Smith and Wakefield, 2015). Athletes commonly use imagery to rehearse skills, regulate emotions (Guillot et al., 2013). Imagery engages psychological and neural processes analogous to those activated during actual performance, thereby enhancing readiness, skill execution, and confidence (Morris et al., 2005). According to Paivio’s (1991) dual coding theory, imagery serves both cognitive functions (e.g., skill acquisition, strategic planning) and motivational functions (e.g., goal setting, confidence building). This distinction has been extended in sport psychology, with Skill and Strategy identified as cognitive imagery dimensions, and Goal, Affect, and Mastery as motivational imagery dimensions (Williams and Cumming, 2011).

Empirical evidence supports the utility of imagery for enhancing Flow. For example, Koehn et al. (2014) developed tailored imagery scripts targeting core Flow dimensions—such as challenge–skill balance, clear goals, concentration, and sense of control—and found sustained increases in Flow experiences among nationally ranked athletes. Similarly, Koehn et al. (2006) reported that sport-specific imagery interventions improved both Flow and performance stability in competitive matches, suggesting that targeted imagery applications can strengthen psychological conditions conducive to Flow in high-performance contexts.

The cognitive and motivational functions of imagery align closely with the conditions required to enter a Flow state, and its effectiveness is enhanced when athletes mentally simulate successful performance aligned with specific goals (Cumming and Williams, 2013). These prerequisites correspond to nine interrelated dimensions empirically linked to superior performance outcomes: challenge–skill balance, Action–awareness merging, clear goals, unambiguous feedback, concentration on the task, sense of control, loss of self-consciousness, time transformation, and autotelic experience (Csikszentmihalyi, 1990). For example, a high challenge–skill balance enhances concentration on the task. This increased concentration promotes deeper flow states and demonstrates how one dimension can influence others (Kawabata and Mallett, 2011). To maximize its impact, imagery should extend beyond replicating technical and tactical elements to incorporate experiential qualities reflecting these nine dimensions. Such dimension-specific and contextually realistic imagery may help create the psychological conditions necessary for Flow (Holmes and Collins, 2002), thereby enhancing both subjective performance quality and objective competitive results.

Despite accumulating evidence linking imagery to Flow, most research has relied on unidimensional assessments (e.g., total scores on the Flow State Scale) or has examined imagery effects on isolated Flow components. Such approaches may fail to capture the multidimensional nature of Flow and may overlook distinct pathways through which cognitive and motivational imagery influence its various dimensions, potentially leading to suboptimal mental training strategies. This limitation may prevent coaches and sport psychologists from designing targeted interventions, ultimately hindering athletes from achieving peak performance through effective Flow facilitation. Moreover, few studies have examined these relationships within an integrated analytical framework capable of simultaneously modeling both the predictors (i.e., imagery types) and the multiple indicators (i.e., Flow dimensions) of the Flow construct.

Building upon existing theoretical and empirical gaps, the present study employs a Multiple Indicators Multiple Causes (MIMIC) model to investigate the direct and indirect effects of five imagery dimensions, as measured by the Sport Imagery Ability Questionnaire (SIAQ), on the nine dimensions of Flow in athletes. A MIMIC model is a form of structural equation modeling that simultaneously estimates the effects of observed predictors on a latent construct and the relationships between the construct and its observed indicators (Kline, 2023). This approach provides a nuanced understanding of how specific imagery abilities may facilitate optimal psychological states in sport performance contexts. By identifying which types of imagery most strongly predict particular aspects of Flow, this study advances theoretical integration between imagery ability and Flow theory and offers practical guidance for designing targeted mental training interventions for athletes.

## 2 Theoretical background and research question

Mental imagery is widely recognized as a multifaceted psychological skill, typically classified into cognitive and motivational functions (Paivio, 1991). In sport contexts, this distinction has been operationalized through Skill and Strategy (i.e., cognitive), and Goal, Affect, and Mastery (i.e., motivational) (Hall et al., 1998; Williams and Cumming, 2011). While both functions are considered important, it remains theoretically ambiguous whether they influence experiential states such as Flow through parallel or distinct pathways. This issue is particularly salient because cognitive imagery often supports preparation and planning, whereas motivational imagery more directly elicits affective arousal, goal commitment, and confidence (Hall et al., 1998; Williams and Cumming, 2011).

Flow, a central construct in optimal functioning research, encompasses multiple dimensions including perceived control, concentration, and autotelic experience (Csikszentmihalyi, 1990). Motivational imagery—by simulating success and reinforcing competence—may align closely with these Flow components (Moritz et al., 1996). In contrast, cognitive imagery may contribute more indirectly by clarifying goals or reducing uncertainty, which subsequently facilitates motivational engagement (Munroe-Chandler and Hall, 2004). Yet, despite these conceptual distinctions, prior research has rarely tested whether cognitive and motivational imagery operate sequentially rather than independently. Instead, most models have treated all imagery functions as direct and equivalent predictors of Flow, often without sufficient theoretical or empirical justification.

Given the current stage of theoretical development, proposing specific directional hypotheses concerning the unique effects of each imagery function on Flow may be premature. The inconsistent empirical evidence and variability in findings across studies underscore the value of adopting an exploratory, research question–oriented approach. This approach enables the investigation of potentially indirect or context-dependent relationships, thereby supporting a more refined understanding of how distinct imagery functions may differentially relate to Flow without presupposing uniform or linear effects.

Importantly, this orientation reflects a theoretically grounded decision rather than a methodological compromise. Both imagery and Flow are inherently complex and multidimensional constructs (Holmes and Collins, 2002), and their interaction is unlikely to be fully captured by narrowly defined or overly deterministic models. By accommodating the possibility of indirect pathways and unanticipated associations, this study remains theoretically anchored while remaining open to alternative structural patterns. This perspective also aligns with broader research suggesting that cognitive strategies, including mental imagery, can be systematically employed to support motivational regulation and enhance performance outcomes (Claver et al., 2017; Fujita et al., 2024).

From an analytical standpoint, the MIMIC model provides a suitable framework for this inquiry. Rather than constraining the analysis to a predefined causal structure, the MIMIC model allows for the simultaneous estimation of both direct and indirect associations while preserving the multidimensional nature of imagery and Flow. This analytic flexibility is advantageous for identifying functional distinctions, mediating processes, and context-sensitive variations. Within this framework, a research question–driven approach offers a theoretically coherent and empirically responsive strategy for advancing understanding in this area. Accordingly, the present study addresses the following guiding question:

RQ. How do cognitive and motivational imagery functions differentially relate to the various dimensions of Flow?

## 3 Materials and methods

### 3.1 Participants and procedure

The present study received approval from the Institutional Review Board (IRB) of the authors’ affiliated university (Waseda University), with the approval number 2020402. Participants were required to be student-athletes officially registered with university-sanctioned sport teams in Japan. Recruitment was conducted through direct outreach by the principal investigator to the athletic departments of participating universities. All prospective participants received a written overview of the study detailing its aims, procedures, and assurances of anonymity and data confidentiality. Participants were informed that their responses would be used solely for research purposes, that participation was entirely voluntary, and that all participants provided written informed consent. All data were collected using a paper-and-pencil method.

Participants provided demographic information, including age, gender, athletic experience, and sport affiliation. At the end of the initial session, they were invited to complete the same scale again after a two-month interval to assess test–retest reliability, given that the short-form dispositional flow measure includes only one item per dimension. After excluding three incomplete responses, which were treated as refusals, the final sample consisted of 375 collegiate athletes (241 males, 134 females; *M* = 19.72 years, *SD* = 1.37). On average, participants reported 11 years of athletic experience (*SD* = 4.14). The sample size (*N* = 375) meets and exceeds recommended guidelines for structural equation modeling, which typically require a minimum ratio of 10–20 participants per estimated parameter (Kline, 2023). Therefore, the current sample is considered adequate for the MIMIC analyses conducted in this study. For the reliability assessment, a subsample of 39 participants agreed to complete the follow-up survey and provided a second set of responses, which enabled the estimation of test–retest reliability.

### 3.2 Measures

Two validated self-report measures were administered to assess athletes’ dispositional flow tendency and sport-specific imagery ability. Athletes’ dispositional tendency to experience flow states was assessed using the Dispositional Flow Scale–2 Short Form (DFS-2; Jackson et al., 2008), a nine-item abbreviated version of the original 36-item scale. Each item represents one of Csikszentmihalyi’s (1990) nine core components of flow: challenge–skill balance, action–awareness merging, clear goals, unambiguous feedback, concentration on the task, sense of control, loss of self-consciousness, time transformation, and autotelic experience (Jackson and Csikszentmihalyi, 1999). Responses were recorded on a 5-point Likert-type scale ranging from 1 (*never*) to 5 (*always*).

Imagery ability was measured using the Japanese adaptation of the Sport Imagery Ability Questionnaire (SIAQ-J; Lee and Horino, 2023), which is a culturally tailored version of the original SIAQ developed by Williams and Cumming (2011). The SIAQ evaluates athletes’ self-reported easiness of generating mental images related to sport. The instrument includes five imagery type—skill, strategy, goal, affect, and mastery—each assessed through three items, totaling 15 items. Participants rated the ease of imagery for each item on a 7-point Likert-type scale ranging from 1 (*very hard to image*) to 7 (*very easy to image*), with higher scores reflecting stronger imagery ability.

### 3.3 Descriptive statistics and bivariate correlations

As shown in Table 1, descriptive statistics were computed for all study variables using SPSS 27.0 to examine central tendency, variability, and distributional characteristics. Shapiro–Wilk tests indicated violations of univariate normality, and Mardia’s coefficient suggested departures from multivariate normality. However, following standard SEM recommendations (Kline, 2023), we additionally evaluated univariate skewness and kurtosis values. All variables fell within the commonly accepted thresholds (|skewness| < 2, |kurtosis| < 7; West et al., 1995), suggesting that the data approximated univariate normality despite significant test statistics.

**Table 1:** Descriptive statistics for imagery ability and Flow dimensions.

Variables	Mean	*SD*	Skewness	Kurtosis
Skill imagery	4.405	1.200	0.056	−0.097
Strategy imagery	4.010	1.291	−0.017	−0.317
Goal imagery	4.630	1.342	−0.164	−0.355
Affect imagery	5.070	1.215	−0.298	−0.093
Mastery imagery	4.589	1.299	−0.204	−0.222
Action–awareness merging	3.211	1.006	−0.258	−0.720
Clear goals	4.024	0.838	−0.785	0.556
Unambiguous feedback	3.117	1.040	−0.151	−0.914
Concentration on task	3.624	0.919	−0.577	−0.014
Sense of control	3.235	0.958	−0.301	−0.761
Loss of self-consciousness	2.845	1.066	0.072	−0.885
Time transformation	3.315	1.058	−0.179	−0.834
Autotelic experience	3.781	0.978	−0.669	−0.011
Challenge–skill balance	4.024	0.900	−0.933	0.616

As presented in Table 2, Pearson’s correlation coefficients between the five imagery abilities and the nine dimensions of Flow ranged from 0.048 to 0.481, all of which were well below the commonly accepted threshold of 0.85 (Tabachnick and Fidell, 2007). These results indicate that multicollinearity was not a concern among the variables included in the analysis.

**Table 2:** Pearson’s correlation coefficients between five imagery abilities and nine Flow dimensions.

Variables	Skill	Strategy	Goal	Affect	Mastery
Action–awareness merging	0.207**	0.210**	0.282**	0.256**	0.384**
Clear goals	0.180**	0.129*	0.398**	0.421**	0.291**
Unambiguous feedback	0.169**	0.276**	0.270**	0.228**	0.410**
Concentration on task	0.178**	0.199**	0.302**	0.409**	0.359**
Sense of control	0.220**	0.190**	0.274**	0.309**	0.437**
Loss of self-consciousness	0.101	0.118*	0.131*	0.048	0.139**
Time transformation	0.152**	0.125*	0.301**	0.343**	0.261**
Autotelic experience	0.146**	0.191**	0.299**	0.481**	0.398**
Challenge–skill balance	0.215**	0.170**	0.369**	0.469**	0.351**

Correlation is significant at the 0.01 (**), 0.05 (*) level (2-tailed).

### 3.4 Measurement model

Measurement validity was examined using a maximum likelihood confirmatory factor analysis (CFA), with model fit evaluated according to recommended cut-off values (Hu and Bentler, 1999; Kline, 2023). A Root Mean Square Error of Approximation (RMSEA) value ≤0.05 was interpreted as indicating close fit, whereas values ≤0.08 reflected reasonable fit (Browne and Cudeck, 1992). For incremental fit indices, Comparative Fit Index (CFI) and Tucker–Lewis Index (TLI) values ≥0.95 were considered to indicate good fit, and values ≥0.90 were deemed acceptable (Hu and Bentler, 1999; Kline, 2023). Standardized Root Mean Square Residual (SRMR) values ≤0.05 represented good fit and those ≤0.08 indicated acceptable fit (Hu and Bentler, 1999). A normed chi-square (*χ*^2^/df) ratio between 2 and 3 was also interpreted as reflecting good model fit (Kline, 2023).

The initial analysis tested the global structure of the DFS-2, in which each Flow dimension was assessed using a single item. The chi-square to degrees of freedom ratio (CMIN/df = 2.78) fell within the recommended range of 2–3, while the CFI (0.973) and TLI (0.955) both exceeded the conventional threshold of 0.95. The RMSEA (0.069) also indicated acceptable model fit. Given the single-item nature of each Flow dimension, AVE could not be computed, and thus Fornell and Larcker’s (1981) criterion for discriminant validity was not applicable. However, the Flow subdimensions were theoretically distinct, and their patterns of association with imagery subscales provided indirect support for construct validity (Diamantopoulos et al., 2012).

As shown in Table 3, temporal stability was assessed using test–retest reliability over a two-month interval. Pearson correlation coefficients for the nine Flow items ranged from 0.625 to 0.885: challenge–skill balance (*r* = 0.885), action–awareness merging (*r* = 0.631), clear goals (*r* = 0.625), unambiguous feedback (*r* = 0.811), concentration on the task (*r* = 0.703), sense of control (*r* = 0.794), loss of self-consciousness (*r* = 0.787), time transformation (*r* = 0.801), and autotelic experience (*r* = 0.782). All coefficients were statistically significant at *p* < 0.01, indicating satisfactory temporal stability. In accordance with Cicchetti’s (1994) guidelines, coefficients exceeding 0.60 are considered indicative of acceptable reliability, supporting the longitudinal consistency of the Flow construct.

**Table 3:** Test–retest reliability of the nine Flow dimensions.

Variables	*r*
Action–awareness merging	0.631
Clear goals	0.625
Unambiguous feedback	0.811
Concentration on task	0.703
Sense of control	0.794
Loss of self-consciousness	0.787
Time transformation	0.801
Autotelic experience	0.782
Challenge–skill balance	0.885

Test–retest reliability coefficients (*r*) for the nine Flow dimensions across a two-month interval.

The five-factor structure of the SIAQ-J exhibited adequate model fit based on multiple CFA indices (see Table 4). Specifically, *χ*^2^ = 189.16, *χ*^2^/df = 2.43, CFI = 0.965, TLI = 0.953, and RMSEA = 0.062. These values meet or exceed conventional thresholds for good fit (CFI and TLI ≥ 0.95, RMSEA ≤ 0.08), as recommended by Hu and Bentler (1999) and Kline (2023). The normed chi-square (*χ*^2^/df) value also fell within the accepted range of 2–3, further supporting the structural validity of the measurement model (Kline, 2023). In the confirmatory factor analysis of the SIAQ-J, all items demonstrated satisfactory standardized loadings, ranging from 0.78 to 0.86 for Skill imagery, 0.79–0.87 for Strategy imagery, 0.63–0.86 for Goal imagery, 0.77–0.87 for Affect imagery, and 0.75–0.89 for Mastery imagery. Collectively, these indicators suggest that the hypothesized factor structure of the SIAQ-J provides a satisfactory representation of the observed data.

**Table 4:** Confirmatory factor analysis fit indices for Flow (DFS-2) and imagery ability (SIAQ-J).

Model	*χ*^2^/df	CFI	TLI	RMSEA
Flow (DFS-2)	2.78	0.973	0.955	0.069
Imagery ability (SIAQ-J)	2.43	0.965	0.953	0.062

Composite reliability (CR) values for the five imagery subscales ranged from 0.756 to 0.869, all exceeding the recommended threshold of 0.70 and indicating acceptable internal consistency (Kline, 2023). In addition, average variance extracted (AVE) values were all above the 0.50 cutoff, with values of 0.650 for affect imagery, 0.679 for skill imagery, 0.681 for strategy imagery, 0.514 for goal imagery, and 0.689 for mastery imagery, demonstrating convergent validity (Fornell and Larcker, 1981). Because each Flow dimension was assessed with a single DFS-2 item, standard convergent and discriminant validity indices (e.g., AVE, Fornell–Larcker) could not be computed. As single-item constructs preclude such evaluations (Diamantopoulos et al., 2012), validity assessment was limited. Nonetheless, acceptable model fit and strong test–retest reliability provide indirect support for the construct’s structural and temporal adequacy.

### 3.5 Structural model and specification

To examine the structural relationships between athletes’ imagery abilities and their dispositional flow tendency, a MIMIC model was specified. MIMIC models are a specialized form of structural equation modeling that allow for the simultaneous examination of latent constructs through their observable indicators and explanatory variables (Kline, 2023). This approach is particularly appropriate when modeling psychological constructs that cannot be directly observed but can be inferred from measured items and predicted by exogenous factors (Jacobucci et al., 2019).

**Figure 1:**
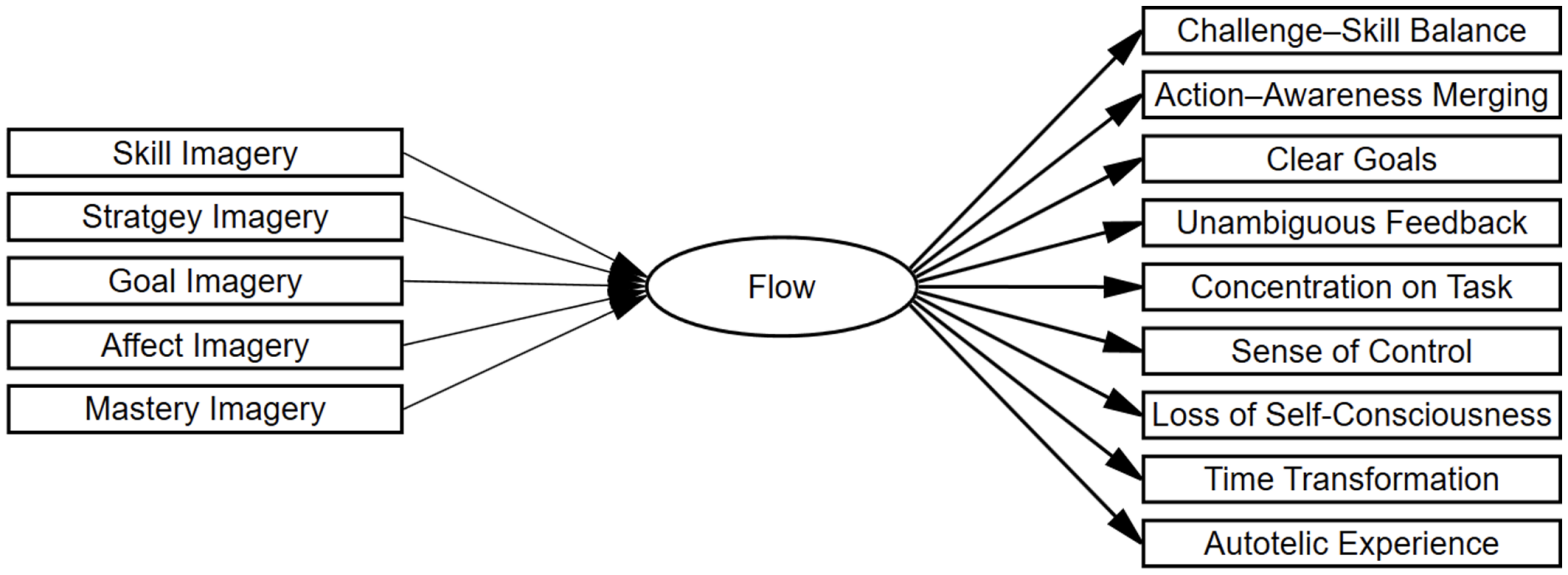
Multiple indicators multiple causes model linking imagery ability (Williams and Cumming, 2011) to nine Flow dimensions (Jackson and Marsh, 1996).

In the present study, the latent Flow construct was modeled using nine observed indicators (one for each dimension), and five imagery ability subscales—Skill, Strategy, Goal, Affect, and Mastery—served as exogenous predictors of Flow. Each predictor was modeled with a direct path to Flow to assess its unique association. Specifically, the initial structural model specified direct paths from each of the five imagery subscales to the Flow construct based on theoretical assumptions (see Figure 1).

**Figure 2:**
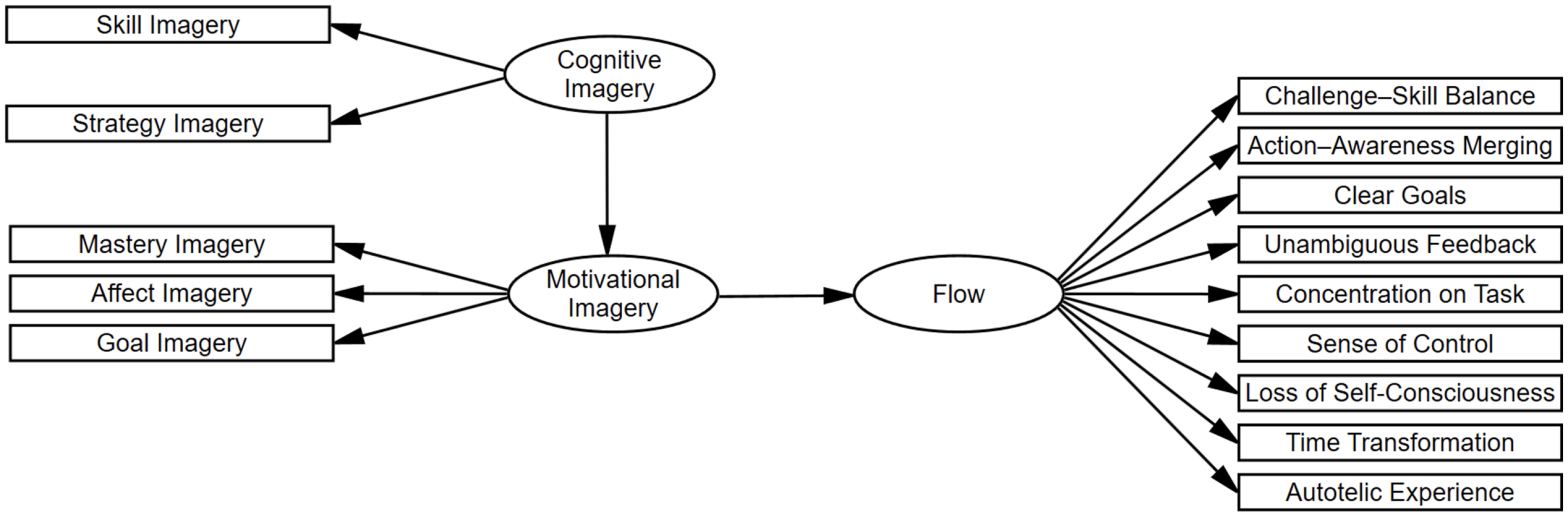
Final alternative model illustrating the mediating role of motivational imagery in the relationship between cognitive imagery and Flow dimensions.

In addition, an alternative model was formulated to examine the possibility that the influence of cognitive imagery (Skill and Strategy) on Flow is mediated by motivational imagery (Goal, Affect, and Mastery). This revised specification was intended to explore the distinct functional contributions of cognitive and motivational imagery in the facilitation of flow experiences among athletes (see Figure 2).

## 4 Results

### 4.1 Initial MIMIC model: direct paths

The initial MIMIC model evaluated the direct paths of five imagery ability subscales—Skill, Strategy, Goal, Affect, and Mastery—on the latent Flow construct. Model fit indices indicated good overall fit: *χ*^2^ = 134.20, *χ*^2^/df = 2.39, CFI = 0.964, TLI = 0.942, RMSEA = 0.061, SRMR = 0.041. Among the imagery predictors, Goal imagery (Estimate = 0.061, *p* = 0.011), Affect imagery (Estimate = 0.160, *p* < 0.001), and Mastery imagery (Estimate = 0.089, *p* < 0.001) demonstrated significant positive relationship with Flow. In contrast, Skill imagery (Estimate = 0.000, *p* = 0.993) and Strategy imagery (Estimate = 0.012, *p* = 0.602) were not statistically significant. These findings highlight a functional divergence between motivational imagery dimensions—which appear to actively facilitate the experience of Flow—and cognitive imagery dimensions, which did not exhibit direct associations. To further elucidate these patterns, standardized indirect effects from each imagery subscale to the nine individual Flow dimensions were computed through the latent Flow factor. As presented in Table 5, motivational imagery subscales yielded consistently positive effects across Flow dimensions, whereas cognitive imagery effects were largely negligible in the direct model.

**Table 5:** Effects of five type of imagery ability variables on Flow dimensions.

Variables	Goal	Affect	Mastery	Skill	Strategy
Challenge–skill balance	0.116 (0.006)	0.275 (0.009)	0.164 (0.030)	0.000 (0.978)	0.022 (0.612)
Autotelic experience	0.113 (0.008)	0.268 (0.009)	0.160 (0.026)	0.000 (0.998)	0.022 (0.578)
Time transformation	0.086 (0.008)	0.204 (0.006)	0.122 (0.028)	0.000 (0.978)	0.016 (0.561)
Loss of self-consciousness	0.024 (0.014)	0.056 (0.010)	0.033 (0.027)	0.000 (0.944)	0.005 (0.400)
Sense of control	0.088 (0.012)	0.209 (0.005)	0.125 (0.026)	0.000 (0.998)	0.017 (0.544)
Concentration on task	0.112 (0.009)	0.266 (0.012)	0.158 (0.034)	0.000 (0.998)	0.021 (0.630)
Unambiguous feedback	0.080 (0.008)	0.190 (0.004)	0.113 (0.015)	0.000 (0.998)	0.015 (0.527)
Clear goals	0.107 (0.009)	0.253 (0.007)	0.151 (0.020)	0.000 (0.978)	0.020 (0.577)
Action–awareness merging	0.081 (0.009)	0.193 (0.009)	0.115 (0.026)	0.000 (0.998)	0.016 (0.511)

Standardized estimates with *p*-values in parentheses; Significance testing for standardized beta coefficients was conducted using 95% bias-corrected confidence intervals. Imagery abilities as an exogenous predictor, and Flow as the endogenous outcome.

### 4.2 Alternative mediation model: indirect pathways

Based on the pattern of results from the initial MIMIC model, an alternative mediation framework was specified to examine whether the effects of cognitive imagery on Flow were transmitted indirectly via motivational imagery. In this revised model, Skill and Strategy imagery were specified as indicators of a higher-order Cognitive Imagery factor, whereas Goal, Affect, and Mastery imagery were specified as indicators of a higher-order Motivational Imagery factor. The model posited that Cognitive Imagery is associated with Flow through Motivational imagery (see Figure 2).

The mediation model demonstrated satisfactory overall fit: *χ*^2^ = 134.20, *χ*^2^/df = 2.39, CFI = 0.964, TLI = 0.942, RMSEA = 0.061, SRMR = 0.041. Standardized path coefficients indicated a significant positive association between Cognitive Imagery and Motivational Imagery (*β* = 0.680, *p* < 0.001), and a subsequent positive association between Motivational Imagery and Flow (*β* = 0.761, *p* = 0.012), supporting a significant indirect pathway from Cognitive Imagery to Flow. To further explore the mediating role of Motivational Imagery, indirect effects from Cognitive Imagery to each of the nine Flow dimensions were estimated. As presented in Table 6, all indirect effects were statistically significant, indicating that Cognitive Imagery influenced Flow across its multidimensional structure via its impact on Motivational Imagery.

**Table 6:** Indirect paths of cognitive imagery on flow dimensions via motivational imagery.

Cognitive imagery ⟹ Motivational imagery	Beta	*p*
Challenge–skill balance	0.577	0.009
Autotelic experience	0.563	0.009
Time transformation	0.428	0.005
Loss of self-consciousness	0.118	0.016
Sense of control	0.439	0.009
Concentration on task	0.557	0.016
Unambiguous feedback	0.398	0.010
Clear goals	0.531	0.006
Action–awareness merging	0.405	0.009

Significance testing for standardized beta coefficients was conducted using 95% bias-corrected confidence intervals. Cognitive imagery is specified as the predictor, motivational imagery serves as the mediator, and Flow as the final endogenous outcome variable.

## 5 Discussion

### 5.1 General discussion

The present study investigated the effects of five distinct imagery abilities—Skill, Strategy, Goal, Affect, and Mastery—on the nine dimensions of Flow using a MIMIC model. The results revealed a clear functional distinction between cognitive and motivational imagery. Specifically, motivational imagery abilities (Goal, Affect, and Mastery) significantly predicted Flow dimensions, whereas cognitive imagery abilities (Skill and Strategy) did not exhibit significant direct effects. Instead, cognitive imagery contributed to Flow indirectly through motivational imagery. The following section offers a detailed discussion of these findings.

Dual coding theory (Paivio, 1991) and the functional classification of imagery (Hall et al., 1998; Williams and Cumming, 2011) offer a theoretical explanation for the pattern observed in which motivational imagery variables (Goal, Affect, and Mastery) directly influenced Flow, while cognitive imagery variables (Skill and Strategy) did not. Within these frameworks, cognitive imagery is primarily associated with skill refinement and technical rehearsal, whereas motivational imagery supports goal orientation, confidence, and affect regulation. The fact that motivational imagery more consistently predicted Flow suggests that imagery promoting emotional engagement and perceived competence may be more effective in eliciting the immersive qualities of Flow than rehearsal-based mental strategies alone.

The findings of our research suggest that motivational imagery may foster Flow by facilitating mental representations of goal attainment, triggering positive emotional states, and reinforcing a sense of mastery. These psychological effects contribute to the internal conditions under which Flow becomes more accessible. The indirect contribution of cognitive imagery—operating through its connection with motivational imagery—suggests a process in which technical or procedural thoughts acquire motivational salience when framed in terms of purpose, affect, or success. This explanation is consistent with prior research highlighting how cognitive strategies can activate motivational resources that enhance sustained performance and engagement (Claver et al., 2017; Fujita et al., 2024).

Treating both imagery and Flow as multidimensional constructs enabled a more nuanced investigation into the differential pathways linking these variables. Rather than assuming a uniform effect of imagery on Flow, the structural model revealed distinct directional patterns across functional categories of imagery. This approach may offer a more ecologically valid perspective on how athletes mentally prepare for performance, and how different imagery functions interact to foster optimal psychological states. Future studies should continue to explore how imagery types contribute uniquely and jointly to constructs such as attention, confidence, or resilience in high-performance contexts.

Patterns identified in the correlational findings further illustrate the differential roles of imagery types (Hall et al., 1998; Williams and Cumming, 2011). Although both cognitive and motivational imagery demonstrated positive associations with Flow, motivational imagery—particularly those related to confidence and affect regulation—appeared to have a more immediate connection with Flow’s experiential qualities. This observation reinforces the idea that motivational imagery is more directly involved in shaping the psychological state of Flow, while cognitive imagery may serve a supportive role by preparing the structure upon which motivational engagement is built. These findings are partially consistent with prior research suggesting that motivational imagery—particularly Motivational General Mastery—exerts a strong mediating effect on Flow (Koehn et al., 2006).

The dimension of Loss of Self-Consciousness revealed a unique limitation in the context of mental imagery. Unlike other Flow components that can be enhanced through deliberate mental rehearsal, the detachment from self-awareness may not be as readily simulated. Imagery, by nature, involves conscious cognitive activity and self-monitoring (Cumming and Ramsey, 2008), which may conflict with the non-reflective absorption characteristic of this particular aspect of Flow. This limitation is consistent with theoretical accounts suggesting that deep immersion and spontaneous attention under real performance conditions are critical for transcending self-referential thought (Csikszentmihalyi, 1990; Holmes and Collins, 2002). Although flow states facilitate a similar reduction in self-referential processing, allowing individuals to transcend self-focused thoughts and achieve a state of optimal performance, this may not always be feasible in real-world settings (Barros et al., 2018). Understanding these nuances can help in developing strategies to harness the benefits of flow and imagery while mitigating its drawbacks.

### 5.2 Theoretical and practical implications

The present findings provide empirical support for integrating Paivio’s (1991) dual coding theory and the functional classification of imagery (Hall et al., 1998; Williams and Cumming, 2011) to explain how different types of imagery contribute to Flow. Dual coding theory posits that mental imagery involves both cognitive functions, such as skill rehearsal and planning, and motivational functions, such as affect regulation and confidence building. The current results affirm this distinction, showing that motivational imagery plays a more direct and proximal role in eliciting Flow, while cognitive imagery contributes more indirectly. Such differentiation validates the theoretical division and emphasizes that Flow is more readily achieved through affectively engaging and goal-oriented imagery content. Moreover, our findings extend this theoretical division by demonstrating that the cognitive and motivational functions of imagery interact hierarchically rather than independently, supporting the dynamic interaction suggested by Cumming and Williams (2013), who argued that imagery functions are most effective when used in an integrated and layered manner.

Beyond validating existing frameworks, this study offers a novel integration by revealing a hierarchical functional interaction between imagery types. Specifically, the finding that cognitive imagery predicts Flow only through motivational imagery suggests a layered psychological process: mental rehearsal may activate emotionally charged motivational content, which in turn enables Flow experiences. This cascade supports a more dynamic understanding of imagery ability, where cognitive and motivational components are not merely parallel processes but sequentially linked. This layered process is in line with the argument that motivational imagery—such as visualizing success or experiencing mastery—is more likely to generate the immersive psychological conditions associated with Flow (Koehn et al., 2014). Future theoretical models should incorporate this mediational logic, particularly when exploring how internal mental simulations translate into complex experiential states such as Flow. As Jackson and Csikszentmihalyi (1999) emphasized, Flow consists of multiple interrelated dimensions, and a multidimensional, structural approach might be essential to understanding how psychological tools like imagery influence each component.

From a practical standpoint, our findings suggest that coaches and sport psychologists should design imagery-based interventions that deliberately integrate both cognitive and motivational components. Rather than focusing exclusively on technical rehearsal (e.g., refining skill execution), training scripts should include vivid and personalized content that emphasizes mastery, emotional engagement, and goal achievement. Supporting this approach, Williams et al. (2013) demonstrated that layering skill-focused and emotionally charged imagery in a sequenced intervention helped an Olympic-level athlete improve both performance and psychological readiness. For instance, a sequence might begin with visualizing proper technique (Skill imagery) and then transition into imagining successful outcomes, feelings of control, and external recognition (Mastery and Affect imagery). This integration may help athletes enter a Flow state more consistently, especially under the pressure of competition.

Additionally, practitioners should be aware that certain Flow dimensions—such as Loss of Self-Consciousness—may not be easily replicated through imagery alone. Since imagery requires active mental effort and self-awareness, it may fall short of producing the complete egoless immersion experienced during live performance. As Munroe-Chandler and Morris (2011) noted, mental imagery is inherently reflective and controlled, making it less capable of inducing spontaneous absorption states compared to real-time sensory engagement. Based on the present findings, imagery can therefore be viewed as a complementary strategy—rather than a substitute for experiential learning—that may help athletes prepare psychologically for situations in which Flow is more likely to arise. When incorporated into pre-performance routines, motivational imagery scripts have been shown to reduce competitive anxiety and elevate confidence levels, thereby improving psychological readiness for Flow (Mellalieu et al., 2009). Therefore, encouraging athletes to rehearse motivational scripts during pre-performance routines may heighten their psychological preparedness, even if not all aspects of Flow can be simulated in advance.

### 5.3 Limitation and future research

Our study is not without limitations, and several factors should be considered when interpreting the results. First, the sample consisted exclusively of collegiate athletes from Japan, with a gender imbalance skewed toward male participants. These characteristics limit the generalizability of the findings across diverse athletic populations. Cultural and gender-based factors can shape the way imagery and Flow are experienced and reported (Gill, 2007). To strengthen external validity, future research should aim to replicate the current findings with more heterogeneous samples, such as athletes from different cultural backgrounds, competitive levels, and with more balanced gender representation.

We employed the short version of the Dispositional Flow Scale. Because the short form does not fully reproduce the original 36-item, 9-factor structure and assesses each Flow dimension with a single item, traditional multi-item reliability indices such as Cronbach’s alpha, composite reliability (CR), and average variance extracted (AVE) cannot be computed. In addition, single-item indicators may yield less stable latent variable estimates. Although we addressed this limitation by examining two-month test–retest reliability to establish the temporal stability of the Flow construct, future research would benefit from developing and employing a multi-item, fully validated 36-item Japanese version of the Flow instrument.

The current study relied entirely on self-report questionnaires to assess both imagery ability and Flow. While these measures are widely used in sport psychology, they are vulnerable to response biases such as social desirability, introspective inaccuracy, and mood effects. These concerns are particularly relevant when studying transient and introspective phenomena such as Flow (Jackson and Marsh, 1996). To improve construct validity and reduce mono-method bias, future studies should consider employing multi-method designs—such as behavioral indicators, observer ratings, or psychophysiological measures—to triangulate self-reported Flow experiences and imagery proficiency (Thomas et al., 2023).

Finally, the cross-sectional design of the present study limits the ability to draw causal inferences. Although the proposed mediation model was theoretically grounded and statistically supported, causal ordering among the variables remains tentative. As Maxwell and Cole (2007) caution, cross-sectional mediation can misrepresent temporal relationships. Future research should employ longitudinal or experimental designs, such as imagery training programs with pre-post assessments or time-lagged designs, to more rigorously test the causal influence of cognitive and motivational imagery on Flow over time and performance outcomes.

## Data Availability

The original contributions presented in the study are included in the article/supplementary material, further inquiries can be directed to the corresponding author.
